# Human metapneumovirus: pathogenesis, epidemiology, diagnostic technologies, and potential intervention strategies

**DOI:** 10.1186/s12985-025-02983-5

**Published:** 2025-11-14

**Authors:** Guicai Gao, Ruihong Lin, Dongli Ma

**Affiliations:** Institute of Paediatrics, Shenzhen Children’s Hospital, Shenzhen, 518026 Guangdong Province China

**Keywords:** Human metapneumovirus, Pathogenic mechanisms, Epidemiology, Diagnostic techniques, Vaccines

## Abstract

Human metapneumovirus (HMPV) is a notable viral pathogen that is responsible for respiratory tract infections in infants, young children, elderly individuals, and immunocompromised individuals. Particularly in the post-COVID-19 era, HMPV has gradually surpassed other respiratory viruses and continues to pose a threat to human health. While substantial progress has been made in understanding the mechanisms of HMPV infection in the host, as well as in terms of diagnostic and prevention methods, no effective vaccines or specific antiviral drugs against HMPV have yet been approved. In this review, we summarize the structure of HMPV and its pathogenic mechanisms; discuss the molecular epidemiology and diagnostic techniques related to HMPV; and summarize the latest advances in the prevention and treatment of HMPV infections, particularly the development of neutralizing antibodies, vaccines, and antiviral drugs. Finally, we discuss the prospects and challenges that lie ahead for HMPV research and clinical interventions.

## Introduction

Respiratory tract infections represent a major global health challenge, consuming substantial medical resources and imposing considerable economic burdens on families and society. Approximately 5 million children younger than 5 years of age were hospitalized because of lower respiratory tract infections (LRTIs) in 2016, with 700,000 fatalities in the same age group attributed to LRTIs [1]. Surveillance data from China covering the period from 2009 to 2019 indicated that the highest positive rate of viral pathogens was observed in children aged 5 years and school-aged children [2]. These infections are caused primarily by various pathogens, including respiratory syncytial virus (RSV), human parainfluenza virus (HPIV), human rhinovirus (HRV), human adenovirus (HAdV), human metapneumovirus (HMPV), and severe acute respiratory syndrome coronavirus (SARS-CoV-2).

HMPV was first isolated and identified in the Netherlands in 2001, with peak circulation occurring in spring, particularly from March to April [2, 3]. It is the second most important viral pathogen responsible for infantile bronchiolitis, following RSV. HMPV manifests with a wide spectrum of clinical presentations, ranging from mild upper respiratory tract infections (URTIs) to severe LRTIs, predominantly bronchiolitis, which can result in hospitalization, serious complications, and long-term sequelae such as wheezing, asthma, and airway hyperresponsiveness in both childhood and adulthood. The clinical symptoms of HMPV infection are often indistinguishable from those caused by RSV [4]. This virus primarily affects children, elderly individuals, and immunocompromised individuals [5]. Among the paediatric population, approximately 10% of hospitalizations are attributed to metapneumovirus [6]. Furthermore, HMPV infection has been shown to increase susceptibility to bacterial superinfection, consequently increasing morbidity and mortality rates [7]. Notably, in 2023, following the gradual spread of the novel coronavirus, HMPV infection emerged as a pandemic in the United States, the United Kingdom, Canada, Spain, and other countries [8, 9].

In this review, we provide a concise overview of the structure of HMPV and the pathogenesis of its infection. We discuss the latest advancements in understanding the host surface receptors that facilitate virus binding. Additionally, we present recent developments in molecular epidemiology and detection techniques associated with HMPV, along with potential intervention strategies.

## The genomic structure of the virion and its mechanisms of pathogenesis

**Fig. 1:**
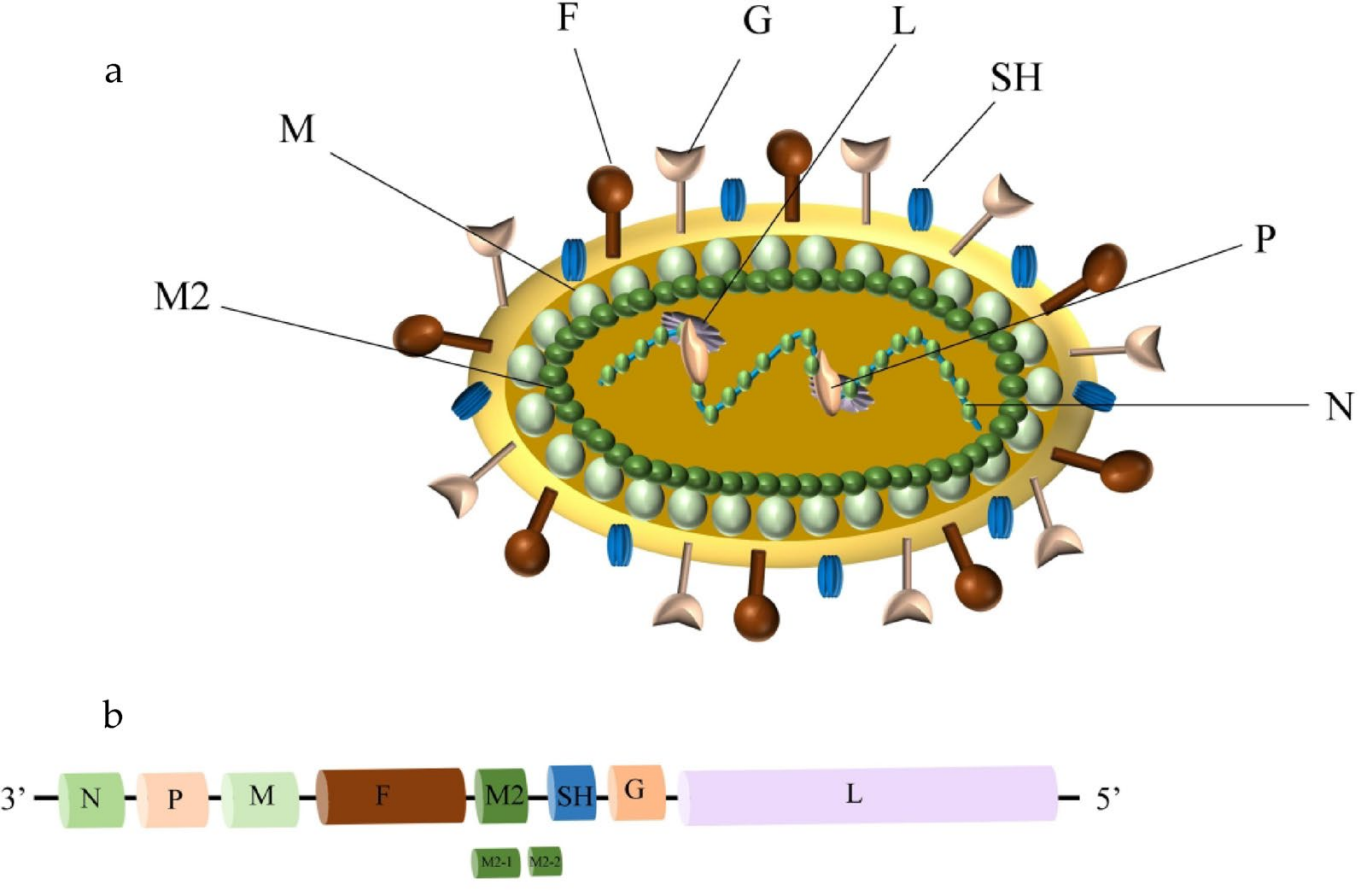
Schematic diagram of the HMPV particle and its genomic organization. (**a**) The general structure of the HMPV and its encoded proteins. (**b**) The genomic organization of HMPV consists of a total of nine proteins: nucleoprotein (N), phosphoprotein (P), matrix protein (M), fusion protein (F), M2-1 protein, M2-2 protein, small hydrophobic protein (SH), attachment protein (G), and polymerase protein (L)

HMPV is a viral particle measuring between 150 and 600 nm and characterized by a short envelope with thickness ranging from 13 to 17 nm. The genome of HMPV consists of negative-sense single-stranded RNA, approximately 13 kb in length, containing eight genes. The genomic organization of HMPV is represented as 3’-N-P-M-F-M2-SH-G-L-5’. These genes encode a total of nine proteins: nucleoprotein (N), phosphoprotein (P), matrix protein (M), fusion protein (F), M2-1 protein, M2-2 protein, small hydrophobic protein (SH), attachment protein (G), and polymerase protein (L) (Fig. 1) [3].

### Surface glycoproteins

The surface glycoproteins of HMPV include G, F, and SH proteins. The G protein, which serves as the primary surface structural glycoprotein of HMPV, is crucial for viral adhesion and is highly genetically diverse [10]. It has been shown to mediate both innate and adaptive immune responses, involving neutrophils, dendritic cells, natural killer (NK) cells, and B cells. Infection with G protein-deficient HMPV is associated with a markedly enhanced T-cell activation phenotype [11]. Furthermore, G protein-mediated innate immune responses may evade NK-cell attacks by downregulating the expression of stress-induced ligands that activate the NKG2D receptor, including major histocompatibility complex (MHC) class I polypeptide-related sequences A and B (MICA, MICB), as well as the UL16 binding proteins ULBP2 and ULBP3 [12]. Additionally, the G protein suppresses innate immune responses in airway epithelial cells by inhibiting TLR4-dependent signalling [13]. Moreover, the G protein regulates the production of neutrophil chemokines and interferon (IFN) responses, specifically IFN-α, by inhibiting the phosphorylation of signal transducer and activator of transcription 1 (STAT1) [14, 15].

The F protein, a major protective antigen among membrane-anchored glycoproteins, has a highly conserved nucleic acid sequence. It plays a crucial role in mediating the fusion between the viral envelope and the cell membrane, thereby establishing viral infectivity [16]. The initial step of HMPV entry into host cells, mediated by the F protein, involves binding to heparan sulfate (HS). After the F protein initially binds to HS proteoglycans, an interaction occurs between integrin and the F protein [17, 18]. F-mediated binding and viral entry rely on the interaction between RGD-binding integrins (such as αvβ1, α5β1, and αv integrins) and the Arg-Gly-Asp (RGD) sequence of the F protein. Notably, the F protein can mediate the binding and fusion of the virus even in the absence of the viral attachment G protein. Thus, the F protein is activated to induce virus–cell fusion through interactions with cellular receptors, and this process is independent of the viral G protein [19–21].

The SH protein, which has viral porin-like properties, influences membrane permeability through its transmembrane domain, resulting in the formation of higher-order oligomers. Notably, the SH protein has been shown to significantly suppress membrane virus fusion activity, a process primarily driven by the F protein. Therefore, it can modulate fusogenic function during viral infection [22]. Beyond its structural role, the SH protein plays a crucial role in evading innate immune responses. Studies have revealed that virus strains lacking the SH protein trigger heightened interferon production in human plasmacytoid dendritic cells (pDCs). Furthermore, the SH protein selectively inhibits the expression of TLR7-dependent genes but does not affect TLR9-dependent pathways. Mechanistically, the SH protein disrupts TLR7/MyD88/TRAF6 signalling, leading to suppressed type I interferon (IFN) gene transcription. Concurrently, it activates the NLRP3 inflammasome, which drives the maturation and secretion of IL-1β, aggravating the inflammation induced by HMPV [23, 24].

### Nucleocapsid

The N, P, and L proteins are central to the transcription and replication machinery of HMPV. The viral genome is encapsidated by multiple copies of the nucleoprotein (N), forming a helical nucleocapsid complex. RNA-dependent RNA polymerase activity resides in the L protein, which binds to the P protein as a cofactor. A critical C-terminal interaction between N and P tethers the L/P polymerase complex to the nucleocapsid, enabling genome replication and transcription. Following viral entry—mediated by glycoprotein-driven attachment, endocytosis, and membrane fusion—the HMPV nucleocapsid is released into the host cytoplasm. Like other nonsegmented negative-sense RNA viruses, HMPV induces the formation of cytoplasmic inclusion bodies (IBs) early in infection. These membraneless organelles serve as concentrated hubs for viral RNA, N, P, and L proteins, facilitating efficient genome replication and transcription [25, 26]. HMPV IBs exhibit remarkable liquid-like organelle properties and undergo dynamic fusion and fission events to optimize viral replication. Previous studies have demonstrated that purified P protein undergoes liquid–liquid phase separation (LLPS) to form condensates in vitro. This differs from other viral systems in which IB formation typically requires multiple viral components. P protein recruits monomeric N protein and N-RNA complexes to these condensates. Through multivalent interactions with N (both monomeric and oligomeric) and RNA, P protein orchestrates IB assembly and stabilizes its liquid-like state [27].

### Matrix protein

The M protein is crucial for shaping viral structure, as it directly facilitates viral assembly and budding. It features two conserved motifs, YSKL and YAGL, with the YAGL motif being particularly important for HMPV assembly [28]. The crystal structure of the M protein in its native dimer reveals a high-affinity Ca2+-binding site and a large positively charged area that interacts with membranes [29]. M protein is taken up by dendritic cells (moDCs) and macrophages, leading to their activation. When exposed to the M protein, moDCs undergo maturation and release various inflammatory cytokines. Additionally, M protein-activated dendritic cells can increase the production of IL-2 and IFN-γ by allogeneic T lymphocytes [30].

**Fig. 2:**
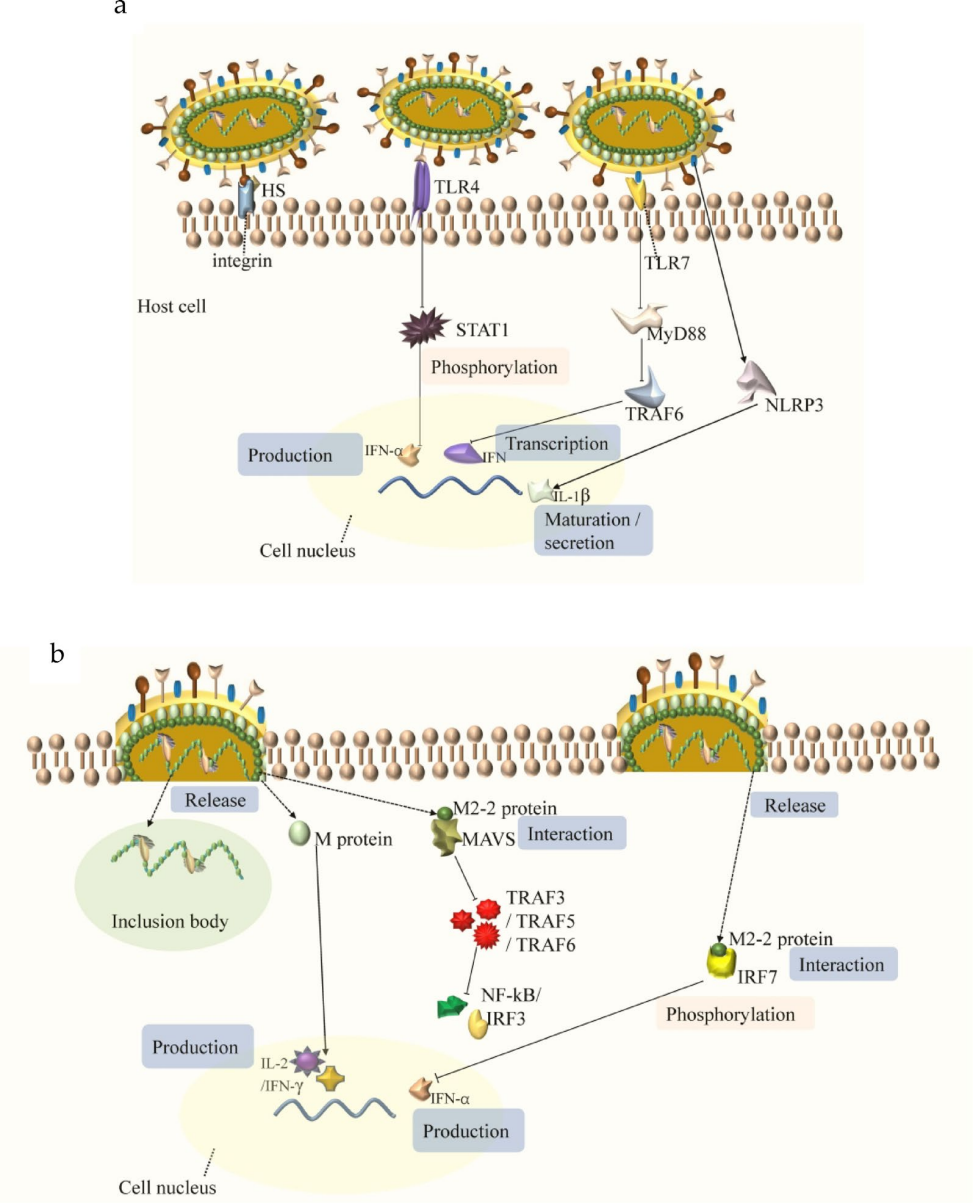
Functions and pathogenic mechanisms of the HMPV protein. (**a**) The surface glycoproteins of HMPV bind to host receptors and regulate signalling pathways. Following initial binding to HS, the F protein interacts with integrin to facilitate cellular invasion. In addition to its adhesion, the G protein can also inhibit the TLR4 signalling pathway, thereby suppressing the production of IFN-α. The SH protein exhibits membrane permeability and exerts pathogenic effects by inhibiting the TLR7 signalling pathway. (**b**) Nucleocapsid and matrix proteins are released into the cytoplasm of host cells and regulate signalling pathways. Viral RNA, N, P, and L proteins form IBs to facilitate genome replication and transcription; M protein stimulates IL-2 and IFN-γ production; and M2-2 targets MAVS to prevent the recruitment of TRAF3, TRAF5, and TRAF6 and corresponding interaction with IRF7 to block IFN-α induction

The M2 gene consists of two overlapping open reading frames (ORFs), M2-1 and M2-2, which are expressed separately. M2-1 is critical for effective viral replication in vivo and functions as a zinc-binding protein that regulates RNA synthesis [31]. The third cysteine (C21) and the final histidine (H25) in the zinc-binding motif (CCCH) of M2-1 are vital for its zinc-binding ability, and a recombinant HMPV lacking this activity shows impaired replication in both the upper and lower respiratory tracts [32, 33]. Conversely, microgenetic analysis has shown that the M2-2 protein inhibits RNA transcription and replication [34]. Furthermore, the M2-2 protein is an important virulence factor that participates in the immune escape of airway epithelial cells by targeting mitochondrial antiviral signalling protein (MAVS) [35]. The PDZ-binding motifs 29-DEMI-32 and 39-KEALSDGI-46 in the immunosuppressive region of M2-2 are responsible for immune evasion, as they prevent the recruitment of TRAF5 and TRAF6, which are downstream adapters of MAVS. While motif 39-KEALSDGI-46 also prevents TRAF3 from migrating to MAVS, M2-2 initiates HMPV immune escape by blocking the interaction between MAVS and its downstream target, TRAF, via its PDZ motif. These TRAFs play important roles in the activation of the transcription factors NF-kB and/or IRF-3 by HMPV [36, 37]. Additionally, the M2-2 protein binds to the inhibitory domain (ID) of IRF7 and prevents its homodimerization by affecting its phosphorylation state, thereby blocking TLR7/9-dependent IFN-α induction [38]. The function and pathogenetic mechanisms of the HMPV protein are shown in Fig. 2.

## Molecular epidemiology

HMPV has been circulating since 1958, but more than 40 years elapsed before it was first isolated and characterized in 2001 [3]. Genotyping of HPMV was performed in 2004, and HMPV isolates clustered in two major genetic lineages, types A and B. Each genetic lineage was divided into two sublineages: A1, A2, B1, and B2 [39, 40].

The A1 genotype of HMPV was no longer detected in the Netherlands after 2006, while other genotypes continue to evolve [41]. This trend appears to be consistent across other countries as well. For instance, the genotypes of HMPV in children with acute respiratory infection from 2007 to 2017 in Vietnam were A2b, A2c, B1, and B2 [42]. In Kenya, from 2007 to 2016, HMPV A2b, A2c, B1, and B2 were identified in 5-year-old children hospitalized with HMPV pneumonia syndrome [43]. Lineages A2b, B1, and B2 have become the dominant strains in China, and lineage A1 has rarely been detected in survey areas [44]. The HMPV genotype A2b was the most prevalent in Ontario, Canada, from 2009 to 2012, with no subtype A1 among the typed samples [45]. In Japan, phylogenetic analysis revealed that subgenome B2 was dominant in 2004, whereas three subgenomes, A2, B1, and B2, cocirculated in 2005 [46]. In Croatia, the A2 and B2 subgroups cocirculated in 2011, 2012, and 2014, whereas B1 was prevalent in 2013 and 2014. Moreover, a new subgroup, A2c, was identified only recently in East and Southeast Asia [47]. In 2017, a sharp increase in the positive rate of HMPV (11%) was detected in Guangzhou, China, which was caused mainly by the HMPV B1 lineage [48]. After the peak of coronavirus disease 2019 (COVID-19), the HMPV-positive samples from children all belonged to genotype B and were divided into subgenotypes B1 and B2 in Hangzhou, China [49].

Notably, since 2014, the HMPV A2b sublineage with a 180-nucleotide repeat in the G gene has emerged as the predominant epidemic strain. In Yokohama city, Japan, HMPV strains were classified into subgroups A2a, A2b, B1, and B2 from 2013 to 2016. Approximately half of the A2b subgroup strains with a 180-nucleotide duplication (180 nt-dup) in the G gene were detected in 2014 [50]. Moreover, genetic variation in HMPV A strains with 180-nucleotide G gene duplication was reported to have circulated in Catalonia from 20142016. Similarly, HMPV had a cycle of 180 nucleotide repeats in the G gene in paediatric patients in the University Hospital of Hebron Valley, Spain, from 2014 to 2016 [51].

**Fig. 3:**
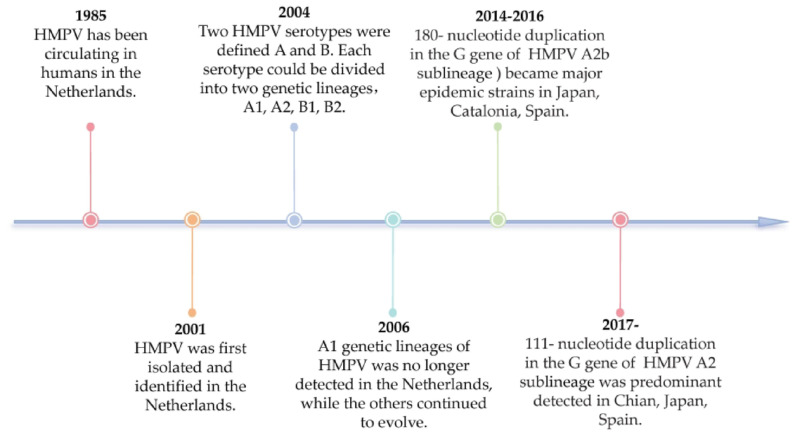
Timeline of the molecular epidemiology of HMPV

However, from 2017 to 2019, novel HMPV subtype A2b strains with 111 nucleotides in the G gene were identified in both Japan and China. These newly identified strains were genetically related to previously recognized HMPV A2b strains with 180-nucleotide repeats [52]. In 2018, the A2b 111 nt-dup strain became the dominant strain in Yokohama, Japan. In contrast, classical HMPV A2b strains have not been detected since 2017 [53–55]. The main time points of the molecular epidemiology of HMPV are listed in Fig. 3 below.

## Diagnostic technologies

Early detection and precise treatment serve as the primary approaches for reducing HMPV infection. Clinically, the main methods for identifying viral infections include in vitro culture, nucleic acid detection, and antigen or antibody detection. However, in vitro culture methods have notable limitations in terms of virus identification. This is because the virus grows slowly in cell culture, which restricts its application in the rapid and efficient identification of viruses. In contrast, nucleic acid detection has emerged as the preferred method for virus identification because of its high accuracy and reliability. Currently available nucleic acid-based detection methods for HMPV include real-time PCR (RT‒PCR), nested real-time PCR, Gexp-based multiple reverse transcription‒PCR, digital microfluidic RT‒qPCR, and CRISPR‒Cas12a.

Taking the N or F genes of HMPV as detection targets, an RT‒PCR method with a limit of detection (LOD) of 100 copies/reaction was established [56, 57]. Researchers have also established a triple single-tube nested RT‒PCR method for the simultaneous detection of RSV, HRV, and HMPV. By targeting the N gene, the LOD for HMPV in this method can reach 5 copies/reaction [58]. Moreover, a GeXP-based multiplex reverse transcription‒PCR (GeXP) assay was developed to simultaneously detect 16 respiratory virus types/subtypes, including HMPV. In this multiplex assay, the L and N genes of HMPV are used as targets, and the detection sensitivity for all premixed viral targets is 1000 copies/reaction [59]. A digital microfluidic RT‒qPCR platform, with the advantages of fewer specimens and ease of operation, has been developed for the detection of 11 respiratory pathogens, including HMPV. By targeting the F gene of HMPV, the LOD of this platform ranges from 12 to 150 copies/reaction [60]. A detection technique combining reverse transcription recombinase polymerase amplification (RT–RPA) with CRISPR-Cas12a using fluorescence or lateral flow (LF) for the clinical diagnosis of HMPV by targeting the N gene was developed, and the detection limit reached 6.97 × 10^2^ copies/mL [61].

Pathogen antigen or antibody detection is a method for detecting the pathogen antigen or the antibodies produced by the host induced by the pathogen. At present, detection methods based on HMPV antigens or antibodies include ELISA, rapid chromatography immunoassay (lateral flow assay), and Luminex bead-based suspension array technology. Sandwich ELISA tests were designed for the detection of hRSV, HMPV, and ADV in human nasopharyngeal swabs, with a LOD of 1 × 10³ pfu/mL [62]. Additionally, two mouse monoclonal antibodies (MAbs) against the N protein of HMPV have been developed and characterized using a lateral flow assay, with LODs of 8 × 10^4^ copies/reaction or 5–10 ng/reaction [63]. Furthermore, the N proteins of HMPV and RSV were also used to develop a duplex assay based on Luminex microbead-based suspension array technology, where diagnostic increases in antibody levels could be determined simultaneously from a single serum dilution. This approach correctly identified most serum pairs with ≥ 4-fold increases in the IgG antibody titre at a single serum dilution [64]. Table 1 shows the different methods for detecting HMPV.

**Table 1:** Characteristics of different methods for detecting HMPV

Detection type	Method	Single or multiple detection	Detection target	Limit of detection	References
Nucleic acid testing	Real-time–PCR	Single and double detection	N gene and F gene	100 copies /reaction	[56, 57]
	Nested RT‒PCR	Triple detection	N gene	5 copies /reaction	[58]
	GeXP assay	Multiple detection(16)	L and N gene	1000 copies /reaction	[59]
	Digital microfluidic RT‒qPCR	Multiple detection(11)	F gene	12–150 copies/reaction	[60]
	RT–RPA-Cas12a	Single detection	N gene	6.97 × 10^2^ copies/mL	[61]
Antigen or antibody detection	ELISA	Triple detection	M protein	1 × 10^3^ pfu/mL	[62]
	Lateral flow assay	Single detection	N protein	8 × 10^4^ copies/reaction or 5 ng–10 ng/reaction	[63]
	Luminex bead-based suspension array	Double detection	N protein	≥ 4-fold IgG antibody titre	[64]

## Potential intervention strategies

Current research highlights several promising strategies to combat HMPV, including antibodies/protein subunit vaccines, mRNA vaccines, live attenuated vaccines, and drug- and siRNA-based therapeutics.

### Antibodies/protein subunit vaccines

Recent studies utilizing high-throughput single-cell technology have identified rare, broadly neutralizing antibodies against the prefusion conformation of the HMPV F protein. These antibodies recognize prefusion-specific epitopes and target the vulnerable site at the tip of the prefusion F trimer. Notably, these neutralizing antibodies demonstrated strong protection against lower respiratory tract infections in small-animal models, positioning them as promising candidates for passive immunoprophylaxis [65]. Studies have further demonstrated that incorporating the M protein into F protein-based vaccines reduces lung inflammation in mice and decreases the Th2/Th1 cytokine ratio among T helper cells. Consequently, the addition of the HMPV M protein to F-based vaccines could modulate both humoral and cellular immune responses to subsequent infection, thereby enhancing vaccine-mediated protection [66]. In addition, several antibody/protein vaccines have entered clinical trials. In a phase 1 clinical trial, the infectivity, safety, and immunogenicity of HPIV3-vectored vaccines expressing the HMPV F protein (HMPV-PreF-A and HMPV-F-B365) were studied in children 24 to < 60 months of age (NCT06546423) in 2024. VXB-241, a molecular clamp-stabilized prefusion F protein subunit bivalent vaccine (RSV preF + HMPV preF) candidate for the prevention of LRTI caused by RSV and HMPV, was launched in phase 1 trials to evaluate its safety, reactogenicity, and immunogenicity in 2024 (NCT06556147). Additionally, Clover Biopharmaceuticals AUS Pty launched a phase 1 clinical trial to evaluate the safety, reactogenicity, and immunogenicity of SCB-1022, which consists of three recombinant protein subunit antigens—the RSV A strain (SCB-1019T(A)), the RSV B strain (SCB-1019T(B)), and HMPV (SCB-1021)—in healthy adults aged 60–85 years (NCT06984094). IVX-A12, an RSV and HMPV bivalent combination virus-like particle protein subunit vaccine, was used in phase 1 studies in older adults aged 60–75 years to evaluate its safety and immunogenicity (NCT05664334).

### mRNA vaccines

A study based on candidate proteins that are strongly associated with viral virulence revealed promising proteins with protective potential: immunodominant cytotoxic T cells, helper T cells, and linear B-cell epitopes were screened from the most promising candidate, the F protein, along with the G, SH, M, and M2 proteins, to construct a multiepitope mRNA vaccine. The results revealed that the vaccine was stable in humans and had the potential to induce strong humoral and immune responses. Fortunately, immune simulations demonstrated that this multiepitope mRNA vaccine was a candidate for controlling HMPV infection [67]. In fact, data have shown that transcribed mRNA encapsulated in lipid nanoparticles is potentially safe and effective in vitro [68–70]. Currently, approximately 5 clinical trials of HMPV mRNA vaccines are registered at Clinicaltrials.gov, including trials of mRNA-1365, mRNA-1653, HMPV mRNA LNP 1, HMPV mRNA LNP 2, and HMPV vaccines (NCT05743881, NCT04144348, NCT03392389, NCT06237296, and NCT06686654, respectively). Among these mRNA vaccines, HMPV mRNA LNP 1, HMPV mRNA LNP 2, and the HMPV vaccine are encapsulated within a lipid nanoparticle (LNP).

### Live attenuated vaccines

The zinc-binding activity of M2-1 is crucial for viral replication and pathogenesis in vivo. Recombinant HMPV lacking zinc-binding activity not only resulted in defective replication in the upper and lower respiratory tract but also elicited strong protective immunity in cotton rats. Thus, inhibition of M2-1 zinc-binding activity could lead to the development of new live attenuated vaccines as well as antiviral drugs for pneumonia [33]. The National Institute of Allergy and Infectious Diseases (NIAID) developed a live attenuated, intranasally administered HMPV vaccine (rHMPV-Pa) and completed phase 1 studies to evaluate its safety and immunogenicity (NCT01255410).

### Drugs

At present, there are no specific drugs for treating HMPV infection. However, according to Clinicaltrials.gov, two nonspecific drugs for treating HMPV infection are currently in phase 2 trials. PUL-042 is in a phase 2 trial evaluating its ability to reduce the severity of lung infections in patients with haematologic malignancies and recipients of haematopoietic stem cell transplantation with documented viral infections due to PIV, HMPV, or RSV (NCT06665100). Another phase 2 study of the nonspecific antiviral drug SNG001 was performed to evaluate the safety, antiviral biomarker responses, and efficacy of SNG001 when it was given to patients requiring invasive mechanical ventilation due to respiratory virus (influenza A (Flu A), influenza B (Flu B), RSV, HRV, HAdV, HPIV, HMPV, or coronaviruses (including SARS-CoV-2 and seasonal coronavirus)) infection (NCT06999603). Table 2 summarizes the clinical trials for intervention strategies for HMPV infection.

### siRNA-based therapeutics

In addition, siRNA targeting the 3’-enriched N and P genes of HMPV resulted in potent sequence-specific viral suppression in vitro. Viral suppression was specific and not mediated by an antiviral interferon-β response or cell death. Thus, siRNAs targeting the N and P genes of HMPV may be sequence specific and highly effective against the virus [71].

**Table 2:** Clinical trials for intervention strategies for HMPV infection

Types	Vaccine name	Clinical status	Clinicaltrials.gov ID	Start date
Protein subunitvaccine	HMPV-PreF-A, HMPV-F-B365	Phase 1	NCT06546423	2024/7/12
	VXB-241	Phase 1	NCT06556147	2024/8/13
	SCB-1022	Phase 1	NCT06984094	2025/6/18
	IVX-A12	Phase 1	NCT05664334	2022/9/21
mRNA-based vaccine	mRNA-1365	Phase 1	NCT05743881	2023/2/15
	mRNA-1653	Phase 1	NCT04144348	2019/11/4
	mRNA-1653	Phase 1	NCT03392389	2017/12/4
	HMPV mRNA LNP 1, HMPV mRNA LNP 2	Phase 1	NCT06237296	2024/1/23
	HMPV vaccine	Phase 1/Phase 2	NCT06686654	2024/11/11
Live attenuated vaccine	rHMPV-Pa vaccine	Phase1	NCT01255410	2011/1/1
Drug	SNG001	Phase 2	NCT06999603	2025/7/1
	PUL-042	Phase 2	NCT06665100	2025/6/27

Sources: ClinicalTrials.gov [72]

## Conclusions and further directions

With the continuous attention devoted to and research conducted on HMPV, the functions of each component of HMPV and its mechanism of interaction with the host have been gradually explored. Researchers have identified several key host cell receptors. In particular, the finding that integrins are functional receptors for F protein-mediated HMPV entry into the host will undoubtedly stimulate many research ideas. Future experiments should directly determine how the F protein interacts with integrins at the molecular structural level. With respect to molecular epidemiology, the A1 subtype of HMPV gradually disappeared, whereas the A2b 111 nt-dup strain gradually became the dominant subtype. These observations give rise to two major questions. Especially after the peak of COVID-19, was the sudden outbreak of HMPV related to the presence of these dominant subtypes? What is the pattern of interaction of these dominant subpopulations with other respiratory pathogens? These questions should be explored in the future. HMPV is a continuous threat to public health, and its epidemiological and genetic evolution characteristics should be continuously monitored. At present, the main methods for identifying HMPV include nucleic acid detection, antigen detection, and serum antibody detection. The sensitivity of nucleic acid detection is high, but it requires professional laboratories, special and expensive instrumentation, advanced professional skills, long detection times, and high detection costs. These drawbacks limit its wide promotion and application. The existing antigen detection sensitivity is low. In the postepidemic era, HMPV has gradually become one of the main pathogens of respiratory tract infection, and there is an increasing demand for home self-testing of pathogens. Point-of-care testing (POCT) can be performed immediately at the sampling site, thereby enabling rapid diagnosis and precise medication for diseases. POCT will help optimize decisions in a timely manner, improve care efficiency, and reduce costs, especially in areas with limited resources. According to the standards determined by the World Health Organization (WHO), an ideal pathogen diagnostic test should be inexpensive, sensitive, specific, easy to use, rapid, and not require large equipment. Rapid POCT conforms to the recommended standards of the WHO and has become an indispensable means for pathogen monitoring, diagnosis, and efficacy evaluation. Therefore, the development of a highly sensitive POCT detection method for HMPV is of significant clinical value. With considerable efforts by researchers to develop HMPV-neutralizing antibodies, vaccines, and antiviral drugs against this pathogen, approximately 12 clinical trials are currently under evaluation. In the next 5 to 10 years, the clinical prevention and treatment of HMPV infection are highly anticipated. The current data concerning the use of neutralizing antibodies, vaccines, or drugs to prevent HMPV are auspicious, and we believe that more than one effective regimen will be approved. However, there are still certain challenges in achieving success with these intervention strategies. For instance, similar to other RNA viruses, surface glycoproteins are prone to mutations. Therefore, for antibody development, it is necessary to identify and monitor the global mutations and distribution of HMPV, as they may affect the results of clinical trials and even the efficacy of future products. Furthermore, the safety and effectiveness of the intervention strategy are crucial for achieving success, and this aspect requires further clarification. Therefore, continuing clinical research on the development of drugs and vaccines for HMPV is of paramount importance.

## Data Availability

Not applicable.

## Abbreviations

HMPV: Human metapneumovirus
LRTI: Lower respiratory tract infections
RSV: Respiratory syncytial virus
HPIV: Human parainfluenza virus
HRV: Human rhinovirus
HAdV: Human adenovirus
COVID-19: Corona virus disease 2019
SARS-CoV-2: Severe acute respiratory syndrome coronavirus
URTI: Upper respiratory tract infections
LOD: Limit of detection
POCT: Point-of-care testing
WHO: World health organization
